# Breeding Wheat (*Triticum aestivum* L.) for Pre-Harvest Sprouting Tolerance in South Africa: Current Status and Future Prospects

**DOI:** 10.3390/plants14142134

**Published:** 2025-07-10

**Authors:** Thobeka Philile Khumalo-Mthembu, Palesa Mmereki, Nokulunga Prudence Mzimela, Annelie Barnard, Toi John Tsilo

**Affiliations:** 1 Germplasm Development, Production Systems & Crop Protection, Agricultural Research Council–Small Grain Institute, Bethlehem 9700, South Africa; mmerekik@arc.agric.za (P.M.); mzimelan@arc.agric.za (N.P.M.); abarnard2812@gmail.com (A.B.); tsilot@arc.agric.za (T.J.T.); 2 Department of Life and Consumer Sciences, University of South Africa, Florida 1710, South Africa

**Keywords:** bread wheat, conventional breeding, modern breeding technologies, pre-harvest sprouting tolerance, South Africa, sustainable development goals

## Abstract

Pre-harvest sprouting of wheat is the premature germination of ripened wheat (*Triticum aestivum* L.) kernels in the spike before harvest and is influenced by a combination of environmental and genetic factors, and their interaction. This greatly affects grain yield and quality, thus posing a threat to food security and sustainable agriculture. Pre-harvest sprouting has been studied for over 30 years in South Africa and remains a trait of interest in our wheat breeding programs amid climatic change. This paper therefore provides a comprehensive review of the progress made, as well as the challenges and limitations encountered, in breeding wheat for pre-harvest sprouting tolerance in South Africa. Future prospects and research directions are also discussed. Conventional breeding has been the main breeding strategy used in the country, with the success of breeding commercial wheat cultivars with durable pre-harvest sprouting tolerance for deployment in the three main wheat production regions of South Africa. Therefore, augmenting conventional breeding with molecular markers and modern genomic breeding technologies is anticipated to speed up breeding locally adapted, climate-resilient wheat varieties that balance tolerance to pre-harvest sprouting with high yield potential. This is key to realizing sustainable development goals of food security and sustainable agriculture.

## 1. Introduction

**Figure 1:**
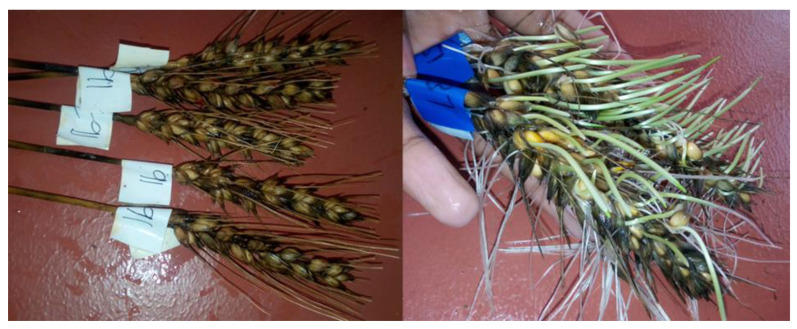
Distinction between pre-harvest sprouting-tolerant (**left**) and -susceptible (**right**) wheat spikes. Symptoms such as discoloration and swelling of the grain, splitting of the seed coat, and emergence of roots and shoots were indicative of pre-harvest sprouting susceptibility [1,39].

Pre-harvest sprouting of wheat is the premature germination of ripened kernels in the spike before harvest caused by prolonged or continuous exposure to wet and humid conditions [1,2,3,4] (Figure 1). The condition not only affects wheat, but other cereal crops as well [5]. This physiological condition is complex and influenced by a combination of environmental and genetic factors, as well as their interaction [6,7,8]. Sprouting results from the activation of enzymes such as lipases, amylases, and proteases inside the grains in response to water absorption, leading to the degradation of lipids, starch, and protein reserves, and thereby increasing the level of gibberellic acid in the seed endosperm [4,9,10,11,12,13,14,15,16]. This greatly reduces grain yield, the baking quality (milling and baking properties) of the produced flour, grain quality, seed test weight, and seed viability and storability, as well as the seedling vigor [1,4,9,10,17,18,19,20,21,22,23,24,25,26,27,28]. As a result, the commercial value of the affected grain is downgraded [1,29,30], and the production and processing of high-quality products are restricted [4,9,10,12,13,14,15,16,24]. Poor-quality foods made from sprouted grains have detrimental effects on human health [5]. Therefore, pre-harvest sprouting is an international concern [6,31,32,33,34,35,36] affecting the entire wheat value chain including producers, grain merchants, millers, maltsters, bakers, other processors, and ultimately the consumer [29]. Annually, pre-harvest sprouting accounts for losses of more than USD 1 billion globally [5,37,38].

The occurrence of pre-harvest sprouting varies across growing seasons due to fluctuations in climatic conditions, thereby complicating the selection and breeding of wheat for pre-harvest sprouting tolerance. With the current unstable global climatic change that is characterized by fluctuations in temperatures and humidity, frequent rainfall, and extreme weather conditions, pre-harvest sprouting and other factors (both biotic and abiotic) compromising grain quality and yield may occur more regularly in wheat producing regions globally [40,41]. This poses a threat to the availability of sufficient high-quality food in the future [42,43].

Pre-harvest sprouting remains a common threat to the South African wheat production and has been extensively studied for over three decades in the country [24,36,44,45,46,47,48,49]. This paper therefore provides a comprehensive review of the progress made and gaps identified in breeding wheat for pre-harvest sprouting tolerance in South Africa. The review (i) covers the significance of pre-harvest sprouting in wheat production and processing; (ii) provides an overview of the South African wheat germplasm and pre-harvest sprouting tolerance; (iii) discusses mechanisms of pre-harvest sprouting tolerance; (iv) outlines breeding strategies for pre-harvest sprouting tolerance; (v) exposes challenges and limitations in breeding for pre-harvest sprouting tolerance; and (vi) suggests future prospects and research directions.

The present study revealed a rich genetic diversity for pre-harvest sprouting in the South African wheat germplasm. Moreover, wheat cultivars and breeding lines with durable pre-harvest sprouting tolerance were identified. Conventional breeding remains the main breeding technique in the country; however, it makes the breeding process lengthy and slow. Therefore, augmenting conventional breeding with molecular markers and genomic breeding technologies is envisaged to facilitate and improve accuracy in breeding wheat for pre-harvest sprouting tolerance in South Africa.

## 2. Overview of South African Wheat Production and Pre-Harvest Sprouting Tolerance

Wheat (*T. aestivum*) is an important staple crop grown in South Africa alongside other crops such as maize, sunflower, and sorghum [50,51]. The crop is planted in approximately 28 of 36 designated crop production regions distributed across the nine provinces of South Africa [52]. According to the 10-year (2014/15–2023/24) analysis of wheat data, the area dedicated to planted wheat amounts to 537,950 ha on average and gives an annual wheat production of 1842 million tons, with an average yield of 3.81 ton/ha [52,53]. Approximately 57% of wheat production occurs under dryland (rainfed) conditions, mainly dominated by the Western Cape (65%) and the Free State (30%) provinces [53]. The remaining portion (43%) of wheat production takes place under irrigation conditions, with the Northern Cape, the Free State, KwaZulu-Natal, and Limpopo provinces accounting for 36%, 23%, 17%, and 9% of the overall wheat production, respectively.

**Figure 2:**
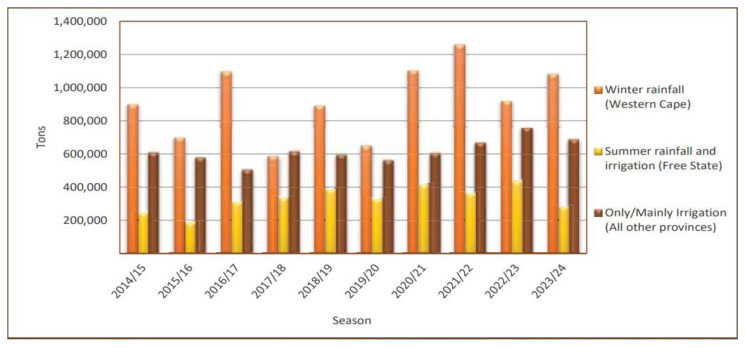
Average yield per production region over the ten-year period (2014/15–2023/24) [52].

The South African wheat production is unique in the sense that there are three distinct wheat production regions, each with their own challenges, specific requirements, and production (yielding) potential [52,54,55] (Figure 2). These include (i) the winter rainfall region, where dryland spring wheat is grown in the Mediterranean climate of the Western Cape province; (ii) the irrigation region, where irrigated spring wheat types are grown adjacent to major rivers in the summer rainfall area; and (iii) the summer rainfall region, where winter and intermediate wheat is sown under rainfed conditions in the Free State province on stored soil moisture accumulated during the preceding summer and autumn. The summer rainfall region receives most of its rainfall in the summer (November–January), during the harvest season for winter wheat [36], and thus it is mostly affected by pre-harvest sprouting.

Pre-harvest sprouting has been a serious concern in South African wheat production, especially for the summer rainfall region. Wheat producers would incur loss at the point of selling their produce due to low (<220 s) falling numbers, but without any symptoms of sprouting [48]. According to the South African bread wheat-grading regulations [56] (Table S1), a cleaned 300 g wheat grain sample must have a minimum falling number value of 220 s and pass a visual inspection tolerance test of not more than 2% sprouted (or damaged) kernels per 25 g wheat sample to be considered free from pre-harvest sprouting damage and classified as Grade 3—Super Grade. Therefore, to assist wheat producers, the ARC-Small Grain formulated a pre-breeding program in the early 1990s, dedicated to screening, developing, and releasing both winter and spring wheat cultivars with improved pre-harvest sprouting tolerance for use in the three main production regions.

Since its inception, the program has evaluated more than 150 commercial wheat cultivars and breeding lines annually for their pre-harvest sprouting tolerance, alongside other important agronomic traits, such as adaptation, grain yield, yield stability, grain quality, resistance to diseases and pests, etc., across the three main production regions of South Africa. Cultivars to be evaluated are provided by the four wheat breeding institutions in the country, namely the ARC-Small Grain, Syngenta Seed (formerly Sensako), Corteva (formerly Pannar Seed), and Limagrain. This has provided South African wheat producers with improved and best-performing cultivars adapted to various environments to choose from based on long-term scientific data [57,58]. As a result, this secured and maximized the yield and quality of the producers’ wheat grain [48,54]. The “then” and “now” pre-harvest sprouting tolerance status of the South African commercial wheat cultivars is summarized in Table 1 below.

Even though the present percentage of cultivars with pre-harvest sprouting tolerance is higher (62%) in the irrigation compared to summer rainfall region (>60%), annual evaluations indicated higher tolerance of rainfed winter wheat cultivars compared to irrigation cultivars [48]. Moderately tolerant or moderately susceptible wheat cultivars are prevalent in the irrigation production region due to the presence of excessive moisture during the later stages of grain filling [59], which renders cultivars vulnerable to pre-harvest sprouting. Therefore, these cultivars are considered sufficient for regulating pre-harvest sprouting and ensuring acceptable falling numbers in this production system [60]. Nonetheless, durable pre-harvest sprouting tolerance has been observed in all three production regions, and cultivars released in recent years showed increased tolerance in contrast to the older cultivars, highlighting the progress made in improving pre-harvest sprouting tolerance in South African wheat [36].

**Figure 3:**
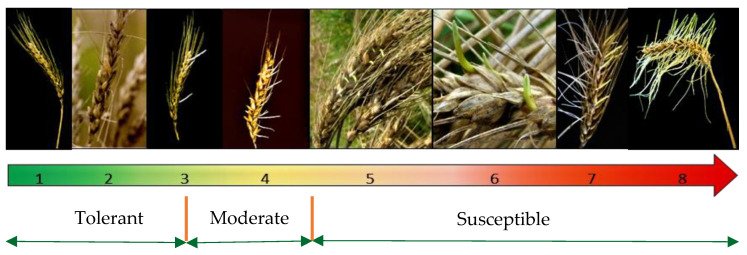
A scale used to screen for pre-harvest sprouting tolerance in the South African wheat, where a score of 1 indicates highly tolerant and 8 represents highly susceptible genotypes [45].

Table 2 below summarizes the rich genetic diversity for pre-harvest sprouting tolerance present in the South African wheat germplasm [36,44,54,61]. The classification was based on a scale of 1 (highly tolerant)–8 (highly susceptible) that is used at the ARC-Small Grain to assess variations in sprouting on intact heads [36,45,54,62] (Figure 3). Moreover, a list of wheat cultivars commonly grown in South Africa over multiple years and seasons, with known pre-harvest sprouting tolerance, is presented in Table S3. The phenotypic and molecular data of these cultivars were obtained through continuous annual evaluations under the ARC-Small Grain’s pre-breeding program for pre-harvest sprouting tolerance in the three production regions and from the research study conducted by [36].

In addition to identified commercial wheat cultivars with good tolerance (Table S3), a set of breeding lines (doubled haploids) with notable pre-harvest sprouting tolerance was also identified by [62,63]. These lines exhibited a combination of low pre-harvest sprouting scores (≤2.99 from the scale of 1–8 (Figure 3)) and high thousand-kernel weight (≥40 g) (Table S4). Few of these lines performed to the level of Elands, the standard pre-harvest sprouting-tolerant parent, with pre-harvest sprouting tolerance scores ≤ 2.00 and thousand-kernel weight of ≥41.6 g. These breeding lines included Tugela-Dn × Elands DH lines nos. 21 and 135 and Elands × Flamink DH lines nos. 9, 44, and 45. This presents a good germplasm recommended for further selection in a pre-breeding or breeding program for pre-harvest sprouting tolerance and yield improvement.

Furthermore, a new research team has been formulated at Stellenbosch University (SU) in partnership with the South African Department of Science and Innovation (DSI), the Technology Innovation Agency (TIA), and Grain SA to navigate the threat of pre-harvest sprouting in the winter rainfall production region of South Africa amidst shifting weather patterns [64,65]. Driven by the increasing incidences of pre-harvest sprouting in the region, this research studies the interaction between the environment and the cultivar’s response to pre-harvest sprouting to make recommendations for cultivar selection based on environmental factors in the Western Cape. Genetic factors and their interaction on cultivars’ response, as well as the impact of management practices such as fungicide applications on pre-harvest sprouting susceptibility, will also be evaluated. This will add value to ongoing breeding efforts aimed at mitigating the threat of pre-harvest sprouting to wheat production in South Africa. No new material has been developed by this team yet. However, the existing genetic resources identified by [36,48,62,63] confirm the genotypic variation available for pre-harvest sprouting tolerance and signify the progress made in breeding for pre-harvest sprouting tolerance in the South African wheat germplasm.

## 3. Mechanisms of Pre-Harvest Sprouting Tolerance

Pre-harvest sprouting tolerance is a complex quantitative trait influenced by a network of developmental, physiological, genetic, and morphological features of the spike and seed [3,6,17,20,29,66,67,68,69,70,71,72,73,74,75,76,77]. Tolerance to pre-harvest sprouting in wheat is predominantly regulated by the seed-dormancy mechanism that favors the production of abscisic acid (ABA) (dormancy-inducing plant growth hormone) over gibberellic acid (GA) (germination-inducing plant growth hormone) [9,71,78,79]. The ABA pathway inhibits the formation of hydrolytic enzymes such as α-amylases, lipases, and proteases in wheat grains [17,80,81], thereby conferring tolerance to pre-harvest sprouting. Several studies have reported the regulatory mechanisms of other hormones, like ethylene, jasmonate brassinosteroids, and auxin, in controlling seed dormancy, germination, and pre-harvest sprouting tolerance [69,70,82,83].

Furthermore, environmental conditions and the developmental stage of the wheat crop have a significant influence on seed-dormancy or tolerance to pre-harvest sprouting [24,64,65,66,84]. Wheat is more sensitive to severity and duration of fluctuations in temperature, relative humidity, and rainfall between grain filling and harvest maturity stages of development, and these conditions are critical in shaping the cultivar’s response to pre-harvest sprouting [1,16,23,29,39,66,67,84,85,86,87,88,89,90]. Higher temperatures and relative humidity, coupled with rainfall, trigger the production of GA in mature wheat seeds, thereby promoting seed germination or pre-harvest sprouting. On the contrary, cooler temperatures and low relative humidity enhance seed-dormancy by increasing the production of ABA and auxin, which are dormancy-inducing plant growth hormones [59,84,91]. Drought-like conditions, coupled with high temperatures, were found to significantly enhance pre-harvest sprouting tolerance in a pre-harvest sprouting-sensitive genotype (white spring wheat variety, Cunderdin) to the levels similar to the well-known dormant genotype (DM2001) [59,85].

Morphological features of the spike and grain also influence seed-dormancy or pre-harvest sprouting tolerance, including spike erectness, spike and awn structure, openness of florets, and seed-coat color and permeability. These features influence the amount of water penetrating through into the seed, as well as the quantity that remains on the spike and the length of time the spike remains wet upon a rainfall event [67,71,77,90,92,93]. Drooped spikes, spikes with awns, and open florets are associated with pre-harvest sprouting susceptibility [64,92], while the impermeable seed coat acts as a physical barrier to prevent water uptake by the zygotic tissues to the germ [94]. Red-colored seeds are known to be more tolerant to pre-harvest sprouting (dormant) than white-colored seeds in wheat [1,95]. This is because of the tight linkage between seed-dormancy genes and seed coat-color genes (R genes) located on group 3 chromosomes of wheat.

Quantitative trait loci and responsible genes associated with seed-dormancy or pre-harvest sprouting tolerance have been identified on all 21 chromosomes of wheat [96]. Global molecular research on pre-harvest sprouting tolerance has uncovered >250 QTLs, and only 29 of these were major and stable across multiple environments [18]. These important (major and stable) genomic regions were identified in various studies [97,98,99,100,101,102,103,104,105,106,107,108,109,110,111]. A summary of major and stable QTLs for pre-harvest sprouting/dormancy-related traits in wheat, as well as their associated molecular markers and physical positions, is given in Table S2. These QTLs are distributed on 12 different chromosomes, namely 1B, 2B, 2D, 3A, 3B, 3D, 4A, 4B, 5A, 6A, 7B, and 7D [18]. Notably, chromosomes from homoeologous groups 3 and 4 harbored 18 of the 29 important QTLs, proving their significance in conferring pre-harvest sprouting tolerance or resistance in various wheat materials around the world [6,17,18,66,112,113,114].

Furthermore, several important genes, including *TaVp-1* [115,116,117,118,119], *TaDOG1L1* [120], *TaMFT/TaPHS1* [27,121], *TaSdr* [93], *TaMKK3* [114], *TaQsd1/TaAlaAT* [122], and *Myb10-D* [9,123], have been mapped and cloned in association with important QTLs for pre-harvest sprouting tolerance in wheat [112] (Table 3). These genes have been used successfully in various studies and have provided effective markers for marker-assisted selection during genetic improvement of pre-harvest sprouting tolerance [9,123]. However, more QTLs, genes, alleles, and pathways associated with pre-harvest sprouting tolerance and seed-dormancy are yet to be discovered and utilized with continuous research. Such information will provide more effective diagnostic markers to expedite breeding pre-harvest sprouting-tolerant wheat cultivars without compromising important agronomic traits, such as grain yield and quality [74].

In summary, the complex and quantitative nature of pre-harvest sprouting tolerance presents both challenges and more opportunities in the improvement of this trait in wheat. Therefore, understanding the influence of and interaction between developmental, physiological, genetic, and morphological features of the spike and seed in regulating pre-harvest sprouting is crucial. Focusing on one promising aspect at a time, i.e., genetic improvement, can be effective and is less influenced by the environment, but it has its own limitations. This underscores the importance of integrating or using a holistic approach with the promise to yield better results.

## 4. Breeding Strategies for Pre-Harvest Sprouting Tolerance

Breeding is imperative for improving wheat productivity and ensuring food security through the development of new wheat varieties that are better-performing, well-adapted to different environments and growing conditions, and possess tolerance to various abiotic and biotic stresses [126]. Various breeding strategies are used to develop wheat germplasm with pre-harvest sprouting tolerance. These strategies vary in their approach and generation time required to achieve a desired outcome [127], but they have been used successfully in different studies conducted in South Africa and across the globe. The first class of these breeding strategies is traditional breeding methods, including phenotypic selection, crossbreeding, backcrossing, mass selection, bulk segregation, evaluation in stressful conditions, and using multi-location trials to identify stable pre-harvest sprouting-tolerant genotypes that perform well under varying climatic conditions [66,112,128,129].

The second class of breeding strategies for pre-harvest sprouting tolerance makes use of modern, more efficient molecular methods such as marker-assisted selection and genomic breeding [128,130]. These include genomic markers [66], genomic prediction models [131], high-throughput genotyping, genome-wide association studies (GWASs) [131], speed-breeding techniques [132,133,134], and gene-editing technologies [112,123,134,135,136,137,138]. The data integration and phenotyping approach also belongs to this class, with the intention to enable a better understanding of pre-harvest sprouting and targeted selection at different growth stages of the wheat crop and under varying environmental conditions. The third class of breeding strategies for pre-harvest sprouting tolerance is called integrated breeding approaches. It embraces the strengths of conventional and modern molecular breeding methods, integrating them to enhance pre-harvest sprouting tolerance effectively [36,113,126].

### 4.1. Conventional Breeding

The significant progress in the development of wheat cultivars with improved pre-harvest sprouting tolerance for deployment in the three wheat production regions of South Africa (winter rainfall, summer rainfall, and irrigation) was achieved through conventional breeding methods [48,54]. The ARC-Small Grain pre-breeding program for pre-harvest sprouting tolerance has been mainly reliant on and continue to use phenotypic selection and testing in multi-locations to identify stable pre-harvest sprouting-tolerant genotypes that perform well under varying climatic conditions of the three wheat-production regions of South Africa. International wheat germplasms with remarkable pre-harvest sprouting tolerance, including AC Domain [101], RL4137 [139,140], Renan [1], Transvaal [141], and Rio Blanco [102], have been crossbred and backcrossed with the South African wheat cultivars possessing various desirable characteristics but lacking pre-harvest sprouting tolerance to obtain improved germplasm. This research work spans three decades in the country, with emphasis on commercial wheat cultivars [24,36,44,45,46,47,48,55], and little research conducted on wheat hybrids [60]. Much of pre-harvest sprouting research work was conducted and documented from the ARC-Small Grain Campus, with a significant contribution from other breeding companies, such as Corteva and Syngenta Seeds. Most of the developed wheat cultivars with pre-harvest sprouting tolerance are shown in Table S3.

### 4.2. Marker-Assisted Selection (MAS)

In contrast to extensive and advanced molecular research conducted on pre-harvest sprouting tolerance in many parts of the world, very limited molecular research work has been conducted and published on pre-harvest sprouting tolerance in the South African wheat germplasm [36,62,142]. For over 30 years, breeding for pre-harvest sprouting tolerance was mainly achieved using the challenging, long, and time-consuming processes of conventional breeding approaches [48,54]. Only recently, wheat breeding programs in the country have incorporated molecular markers in breeding for pre-harvest sprouting tolerance to facilitate the efficient identification and targeted selection of breeding lines with pre-harvest sprouting tolerance (genes and alleles) [48,62,142].

One study [36] demonstrated the potential of molecular markers to expedite screening and selection of pre-harvest sprouting-tolerant varieties to use in breeding stable pre-harvest sprouting-tolerant genotypes that are adapted to the South African environment. The study identified favorable haplotypes for major QTLs regulating pre-harvest sprouting tolerance located on chromosomes 3A and 4A that explained >40% of the observed phenotypic variance in the South African wheat germplasm. The presence of different allelic versions of candidate genes or the presence of novel mutations at the 3A and 4A QTL regions was speculated and requires further validation. It is presumed that every new study on the genetics of pre-harvest sprouting leads to the identification of few novel QTLs or marker–trait associations (MTAs), indicating the deeper genetics of pre-harvest sprouting tolerance and an existing potential for discovery of new loci [131].

Furthermore, ref. [36] demonstrated that the cultivar’s phenotype class could be predicted with accuracy and efficiency using the simple sequence repeat (SSR) marker haplotyping; however, the prediction accuracy improved with the advanced (Kompetitive Allele-Specific PCR) molecular technology, proving the suitability of the methodology in future pre-harvest sprouting screenings. Predicting the cultivar’s pre-harvest sprouting tolerance class was the very first attempt in the South African wheat commercial cultivars. In a different study [62], single-nucleotide polymorphism (SNP) and silicoDArT markers were deployed to uncover three stable QTLs with major effects (PVE = 10.08–20.30%) on chromosomes 5B and 7B, regulating pre-harvest sprouting tolerance in a DH wheat population adapted to the South African environment. These studies [36,62] summarize the molecular research that has been conducted and published to date on pre-harvest sprouting tolerance in the South African wheat germplasm.

### 4.3. Genomic Selection

Genomic selection and gene editing have come out as powerful tools known to accelerate breeding for complex traits like pre-harvest sprouting tolerance [143,144]. There are no published records for the application of these techniques in breeding for pre-harvest sprouting tolerance in the South African wheat germplasm; however, notable achievements about the improvement of various complex quantitative traits in wheat through genomic selection and gene editing have been reported in other countries. Genomic prediction (GP) has been used successfully in combination with genome-wide association studies (GWASs) to uncover 26 pre-harvest sprouting-responsive genomic regions spread over 16 chromosomes of wheat based on sprouting score, falling number, and grain color [131]. About 20 definitive and stable quantitative trait nucleotides were considered important for use in marker-assisted recurrent selection. The study [131] also reported higher comparable genomic prediction accuracies (0.41–0.55) for the three sprouting traits. Another example of genomic selection includes the successful transference of a maize seed-dormancy gene *Vp-1* (including promoter and coding regions) into the wheat variety Zhengmai 9023, which significantly reduced seed germination index values in the transgenic generations (T3 = 79%, T4 = 80%, and T5 = 82%) compared with the wild type [145].

Advances in omics technologies have further enabled the identification of causal genes for breeding, although functional validation remains challenging due to inefficient transformation protocols [136]. Clustered regularly interspaced palindromic repeats-associated protein 9 system (CRISPR-Cas9) is a genome-editing technique that has been successfully used for plant functional gene research and crop genetic improvement in wheat and other cereal crops [134]. The technique can precisely target any gene of interest using engineered site-specific nucleases and remove, insert, or mutate a DNA sequence [146,147]. For instance, CRISPR-Cas9 technology was successfully used to improve pre-harvest sprouting tolerance in wheat by editing the seed-dormancy locus *TaQsd1*, which resulted in genetically edited wheat with significantly longer dormancy period compared with the wild type [135].

In recent findings, the technology restored *Tamyb10* in the white-grained variety Fielder to create pre-harvest sprouting-resistant wheat [138]. The resultant transgenic plants with edited *Tamyb10-B1a* changed seed coat color from white to red, in addition to enhanced anthocyanin index of seed and pre-harvest sprouting resistance compared to the wild type (Fielder). The most recent developments in this work further validated the association of *TaMyb10* homologs with grain color and seed germination in wheat. Accessions exclusively harboring *TaMyb10-D* displayed red seed-coat color and reduced germination percentages, indicating the predominant role of *TaMyb10-D* compared to *TaMyb10-A* and *TaMyb10-B* [123]. These results demonstrate the potential of gene-editing technology to effectively improve breeding progress of pre-harvest sprouting resistance in wheat [112,137]. Importantly, gene-editing technologies can also reverse the loss of key traits that occurred during wheat domestication [137].

### 4.4. Integrated Approaches

Integrated breeding approaches that combine conventional methods with modern molecular tools have been commonly used to address the complex genetic and environmental factors influencing pre-harvest sprouting tolerance [112,131]. For example, integrating genomic-assisted breeding and genome-editing tools with speed-breeding technology offer the potential to accelerate selection, boost genetic gains, and transform PHS breeding efficiency in both global and South African contexts [132,133]. One study [74] proved the strength of integrated breeding through combining field trial evaluations with SNP markers to identify PHS-tolerant lines without compromising flour quality or the agronomic value of soft winter wheat. The current and future research on pre-harvest sprouting tolerance in the South African wheat germplasm aims to further explore the power of combining conventional breeding with marker-assisted breeding [36,62] and genomic breeding to expedite wheat improvement.

## 5. Challenges and Limitations in Breeding for Pre-Harvest Sprouting Tolerance

Climate change remains the main challenge in breeding wheat for pre-harvest sprouting tolerance. The shifting weather patterns associated with climate change exacerbate pre-harvest sprouting, thus presenting a huge threat to the availability of enough and high-quality food in future [13,42,43,66,148,149]. Recently, there has been an increase in the prevalence of pre-harvest sprouting in wheat-producing areas of South Africa [52,64,150,151,152] and other parts of the world [34,66,153]. This can be attributed to the unpredictable and heavy rainfall patterns occurring at physiological maturity, coupled with the fluctuation in temperature and humidity levels throughout the growing season, which break seed-dormancy and thus make wheat varieties more vulnerable to sprouting [13,62,64,66,125]. These conditions may be expected to occur very often in the future [40,41], greatly reducing the quality and yield of the produced wheat grain [154].

Genetic bottlenecks such as the limited genetic diversity imposed on wheat during domestication and in modern breeding practices and targeted selection present another challenge when breeding for pre-harvest sprouting tolerance [66,154]. This indicates the need to create new genetic diversity through genetic-improvement studies (i.e., mutation breeding) and germplasm exchange, as well as using landraces and wild relatives of wheat [6,19,137,154,155]. New genes, QTLs and alleles conferring pre-harvest sprouting tolerance can be mined, discovered, and deployed to develop improved wheat varieties [13,75,110,124,156]. Moreover, wild relatives, landraces, and wheat varieties with remarkable pre-harvest sprouting tolerance present valuable sources of genetic diversity for pre-harvest sprouting tolerance and adaptation to local environmental conditions. Therefore, introducing genes from these sources would not only enrich the genetic base for pre-harvest sprouting tolerance in modern wheat breeding programs, but would also facilitate the development of wheat varieties with improved wheat grain yield, quality, pre-harvest sprouting tolerance, and adaptation to local environmental conditions [66,154,157]. Table 4 below lists exotic landraces, varieties, and wheat breeding lines exhibiting pre-harvest sprouting tolerance, which have been identified and successfully used in different wheat breeding studies [66].

Another challenge or limitation in breeding wheat for pre-harvest sprouting tolerance is the complex nature of pre-harvest sprouting. Pre-harvest sprouting is a complex trait that is influenced by numerous other traits and factors [3,6,68,69], implying that a pure selection targeted at improving pre-harvest sprouting tolerance will impact other traits as well. Therefore, understanding correlations between pre-harvest sprouting tolerance and grain yield, yield components, grain protein components, and other important agronomic traits is of paramount importance for wheat breeding programs to enable thoughtful and necessary selections [41,167]. It is documented that selection targeted at individual loci will reduce genetic diversity within and around the selected loci [168].

The negative association between pre-harvest sprouting and grain quality limits the production of high-quality wheat grains. Pre-harvest sprouting negatively impacts grain quality by reducing the baking and cooking properties of the produced flour [1]. The reduction in grain quality is due to the degradation of starch and protein in germinated kernels by alpha-amylase activity and is shown by the reduction in Hagberg falling number [41,169]. Flour and dough made from sprouted grains produce bread products with darker crust, sticky crumb texture, and poor slicing ability [1,80,170,171,172,173]. Moreover, the breakdown of grain carbohydrate reserves adversely impacts the quality of the cooked pasta and the malting process [23,174]. Poor-quality foods made from sprouted grains have detrimental effects on human health [5].

Pre-harvest sprouting also negatively impacts grain yield. Highly tolerant or dormant wheat seed results in tardy, variable, and poor seed germination and seedling establishment, which negatively affect grain yield [167]. Direct and indirect effects of pre-harvest sprouting on grain yield and yield components were evaluated in the Brazilian wheat germplasm using eight cultivars known to possess high pre-harvest sprouting tolerance [167]. Pre-harvest sprouting exhibited highly significant and positive genotypic (r = 0.752) and phenotypic (r = 0.716) correlations with grain yield, which indicated lower pre-harvest sprouting tolerance (susceptibility) in wheat cultivars with higher grain yield and productive potential. The path analysis further confirmed the highest direct effect (r = 0.511) of pre-harvest sprouting on grain yield, indicating that cultivars with less tolerance to pre-harvest sprouting have the highest grain yield in wheat [167]. The study also observed a reduced effect of pre-harvest sprouting on plants with shorter stature and lower hectoliter weight. The findings of this study [167] concur with the observations of [175], who still found no cultivar with both high grain yield and good tolerance to pre-harvest sprouting, despite using different wheat cultivars.

On the contrary, two studies [165,176] evaluated and identified wheat genotypes and combinations thereof with the potential of high tolerance to pre-harvest sprouting and grain yield. This underscores the possibility of achieving both pre-harvest sprouting tolerance and high yield potential in a single wheat variety, which is a much sought-after combination in wheat breeding programs worldwide [165,176]. However, this comes as a result of a calculated compromise: selecting for seed with intermediate dormancy level and high productive potential (i.e., high number of spikes per square meter) to achieve good plant establishment and ultimately higher grain yield in the field, while providing a good level of tolerance to pre-harvest sprouting to secure grain quality [41,69,167]. Moreover, molecular markers present an opportunity to rapidly characterize diverse germplasm for pre-harvest sprouting tolerance sources and transfer them to the well-adapted and high-yielding cultivars [132,177].

## 6. Future Prospects and Research Directions

Future prospects and research directions in breeding wheat for pre-harvest sprouting tolerance will be guided by three main themes, namely climate-resilient breeding, technological advances, and collaborative initiatives and policy support. These themes are critical in enhancing the ability to produce wheat varieties with tolerance to pre-harvest sprouting that adapt well to adverse conditions (changing climate) and maintain high productivity. Such is pivotal in attaining food security and sustainability.

### 6.1. Climate-Resilient Breeding

Wheat remains a vital food crop that nourishes billions of people worldwide [137,178,179,180,181]. However, factors such as climate change and the rapid population growth exert pressure on wheat production, necessitating a double increase in production to meet the demand and attain sustainability [40,42,43,148,149,179,182]. The impacts of climate change, such as erratic climatic fluctuations, are associated with an increase in pre-harvest sprouting incidences in wheat-growing regions globally. This greatly reduces grain yield and quality, lowers the producers’ income, and is associated with significant annual economic losses [5]. With the projected increase in temperatures, gradual population rise, and stagnant or reduced wheat yields despite substantial breeding efforts [183,184], breeding for climate-resilient wheat genotypes is both a necessity and a priority for global food and nutritional security [137,185]. This aligns well with the Sustainable Development Goal 2.0 (SDG 2.0) of the United Nations Agenda 2030 and its set targets [128,178,186,187,188,189], which aim to eradicate all forms of hunger and malnutrition worldwide by 2030, in addition to promoting sustainable agriculture [128,178,187].

For a climate-resilient breeding program with the intention to improve grain yield and quality or pre-harvest sprouting tolerance, the focus should be on genotype, environment, and genotype × environment interactions [190]. Priority traits should include intermediate seed-dormancy, early maturity, extended booting-to-heading and booting-to-anthesis durations, shorter heading-to-anthesis duration, selection of shorter genotypes for irrigated environments, and taller genotypes for heat stress and heat–drought-prone environments [178]. In terms of morphological traits, erect spikes with awns or epicuticular waxes (umbrella effect), harder kernels, and impermeable and red seed coats should be selected to reduce the negative impacts of pre-harvest sprouting [65,73].

Developing and deploying pre-harvest sprouting-tolerant wheat varieties that can withstand future climate conditions is a more sustainable, economical, safer, and effective approach to reducing damage caused by pre-harvest sprouting [17,89,112,137]. This can be achieved through identification and introgression of major dormancy genes and alleles from the wild wheat relatives and landraces to improve the adaptability and productivity of modern wheat varieties under changing climates [16,25,74]. For example, *Aegilops tauschii* (the D genome donor of bread wheat) is characterized by high variation for pre-harvest sprouting tolerance [104,158,191] and is a good source of pre-harvest sprouting tolerance [192]. Three *Ae. tauschii*-derived dormancy QTLs, namely *QDor.3D.1*, *QDor.3D.2*, and *QDor.3D.3*, were identified on the long arm of chromosome 3D, using four SSR markers [157]. The study further developed 10 Kompetitive allele-specific PCR markers that will expedite map-based cloning of these loci and their introgression into bread wheat [157].

Another approach to developing pre-harvest sprouting-tolerant wheat varieties that can withstand future climate conditions is using improved cropping systems. This involves shifting planting dates to enable harvesting outside of the rainfall season and using seasonal/regional weather forecast to predict the pre-harvest sprouting risk. Tolerance to pre-harvest sprouting depends on the cultivar choice (genotype), the developmental stage, the amount and duration of rain, the temperature level, and the moisture content, as well as the interactions between these factors [64,65,66,71,74,84]. Therefore, ref. [65] suggested that seasonal or regional weather forecast information may be used to advise producers on when to plant and harvest and which cultivar choice will be suitable for their production regions given the prediction of environmental conditions for the season.

Understanding the relationship between pre-harvest sprouting tolerance and other traits of agronomic importance is also crucial in the development of pre-harvest sprouting-tolerant wheat varieties that can withstand future climate conditions. Despite the negative association between pre-harvest sprouting tolerance and grain yield, as well as quality, ref. [74] recently reported the opposite. This study [74] reported the possibility of improving pre-harvest sprouting tolerance in wheat under adverse weather conditions brought about by climate change without affecting flour quality or the agronomic performance of soft winter. Other approaches to develop pre-harvest sprouting-tolerant wheat varieties that can withstand future climate conditions may include exchanging germplasm between countries and breeding programs; improving farm policies; investing in breeding wheat varieties that are climate-resilient and possess pre-harvest sprouting tolerance; and deploying advanced breeding techniques, such as gene editing, speed breeding, and other modern breeding technologies.

### 6.2. Technological Advances

Harnessing the technological advances and scientific progress made up to date is essential for better management of pre-harvest sprouting [137]. Such technological advances in genetic and biotechnological research enhance our understanding of the mechanisms involved (i.e., seed-dormancy) and genetic factors associated with tolerance to pre-harvest sprouting. This enables breeding environmentally adapted wheat varieties with acceptable pre-harvest sprouting tolerance [69,137,167].

Complimenting conventional phenotyping techniques with the use of molecular markers for selection, as well as high-throughput screening technologies, has improved breeding efficiency. This has been validated by a few studies conducted on South African wheat [36,62], as well as numerous studies conducted across the globe [66,113,131]. Traits or genomic regions associated with pre-harvest sprouting tolerance were identified and successfully introgressed from good sources of tolerance (genotypes such as Frontana, Grandin, ND 674, and RL 4137) into locally adapted and high-yielding germplasms [156], using both conventional (backcrossing) and modern breeding techniques [66,113]. The efficiency of molecular markers in MAS was proven by [113] to expedite crop improvement by successfully stacking multiple loci of agronomic importance, including pre-harvest sprouting tolerance, grain protein content, and leaf rust resistance, into a single plant.

The identification of several genes underlying major QTLs regulating pre-harvest sprouting or seed-dormancy has also enabled the manipulation of these genes, using various approaches such as CRISPR/Cas9 technology to enhance pre-harvest sprouting tolerance in wheat. The gene-editing technology CRISPR-Cas9 has yielded significant progress in breeding wheat varieties with pre-harvest sprouting tolerance [112,123,135,136,137,138]. Through this technology, important seed-dormancy genes that were lost or silenced through domestication are being restored or edited [137]. Moreover, new wheat varieties with desired variations in pre-harvest sprouting tolerance are developed through silencing genes associated with seed germination or related pathways.

The integration of genome editing tools, genomic-assisted breeding, and speed-breeding technology presents possibilities to further enhance breeding efficiency by increasing selection accuracy and genetic gain per year [132,133]. These results demonstrate the potential of modern breeding techniques to expedite breeding for pre-harvest sprouting and highlight promising prospects for future research in the South African wheat industry.

### 6.3. Collaboration and Policy Support

Pre-harvest sprouting is a global issue, and policies have been put in place to support efforts aimed at mitigating the negative effects of pre-harvest sprouting on the countries’ economies, food security, and the environment through breeding for pre-harvest sprouting tolerance. These include the use of molecular breeding techniques [17,18,66,112], phenotypic selection [193], identification of tolerance sources and tolerance genes [22,66,112], and combining breeding techniques with management practices [65,137].

Supporting policies related to breeding wheat for pre-harvest sprouting tolerance and encouraging collaborations between countries and breeding programs working on understanding pre-harvest sprouting are efficient ways to drive future research. Engagement between breeders, producers, researchers, and policymakers can improve the conducted research and strengthen breeding programs. The integral role and benefits of international collaborations and policy support were demonstrated during “The Green Revolution” era [194,195] and afterwards, leading to the economic development and improvement of food security in many countries.

South Africa does not have enough capacity (resources and skills) to conduct the latest cutting-edge genomic research on pre-harvest sprouting, thus hindering the progress in this field. Therefore, collaborating with countries and research laboratories that are leading in this space will build up the much-needed capacity, enable knowledge and germplasm exchange, and open doors to funding opportunities. For example, South Africa is working closely with international agricultural research centers such as the International Maize and Wheat Improvement Centre (CIMMYT), the International Centre for Agricultural Research in the Dry Areas (ICARDA), and the United States Department of Agriculture (USDA) to improve its wheat germplasm and farming systems, as well as addressing issues related to climate change and food production. These collaborative initiatives have resulted in the enhancement of genetic diversity in our modern wheat varieties and contributed significantly to the improvement of the country’s food security. It is reported that most of modern wheat varieties contain at least one CIMMYT parent in their pedigrees [196]. Moreover, the pre-harvest sprouting tolerance of most South African cultivars traces back to similar sources in Canadian and Australian wheat cultivars [61], demonstrating the significant role played by international germplasm in supporting local breeding efforts.

Recently, South Africa participated in the latest (15th) International Symposium on Pre-Harvest Sprouting in Cereals (ISPHSC) held in Tsukuba, Japan, on 4–6 October 2023. The ISPHS is held once every three to four years and offers a platform for researchers, breeders, and seed companies from all over the world working on understanding pre-harvest sprouting using multidisciplinary approaches to gather and share/discuss recent developments (i.e., generated knowledge, developed germplasm, etc.) in breeding for pre-harvest sprouting tolerance in cereals. The status and future prospects in breeding for pre-harvest sprouting tolerance in the South African wheat germplasm were shared, and new knowledge was gained from the symposium presentations. Moreover, potential collaborations between South Africa and Japan, as well as between South Africa and China, were identified. Japan and China have conducted extensive and advanced molecular and genomic research on pre-harvest sprouting tolerance in wheat and other crops of agronomic importance [9,17,27,31,32,112,123,138,160,197,198,199,200,201,202]. Therefore, collaborating with these countries could be very beneficial and enhance the research conducted on the South African wheat germplasm.

The integrated approach of collaborations and policy support, technological advances, and the development of climate-resilient wheat germplasm that is tolerant to pre-harvest sprouting will enable a better understanding and manipulation of the genetic diversity for pre-harvest sprouting tolerance existing in our own wheat germplasm. This will facilitate the development of well-adapted wheat cultivars with intermediate tolerance to pre-harvest sprouting and high yield potential, contributing to both economic and environmental benefits.

## 7. Conclusions

Breeding wheat for pre-harvest sprouting tolerance is important for the development of climate-resilient varieties. This is necessary to protect grain yield and quality, and to reduce economic losses associated with pre-harvest sprouting, thereby improving food and nutritional security around the world. The South African wheat germplasm contains a rich genetic diversity for pre-harvest sprouting tolerance, which can be explored with the assistance of molecular markers and genomic tools. Moreover, wheat cultivars with durable pre-harvest sprouting tolerance have been developed and released into the market. Tolerant wheat germplasms have also been reported in other countries and can be accessed through germplasm exchange.

Despite all the developments in identifying and developing wheat germplasm with pre-harvest sprouting tolerance and the advancements in wheat genome sequencing, the progress in breeding for pre-harvest sprouting tolerance in the South African and global wheat germplasm is slow. This could be attributed to a number of factors, including (i) the lack of or limited capacity (resources and skills) allocated to the improvement of pre-harvest sprouting tolerance/seed-dormancy in wheat; (ii) the polygenic nature of the trait; (iii) poor understanding of pre-harvest sprouting/seed-dormancy and non-standardization of phenotyping techniques; (iv) trade-offs between pre-harvest sprouting tolerance and other agronomic traits of importance, such as grain yield; (v) limited available diversity in germplasm for the trait, which necessitates identification of newer sources of pre-harvest sprouting tolerance; (vi) the lack of suitable donors with seed-dormancy; (vii) breeding strategies used in breeding programs (most of the progress made is attributable to conventional breeding); (viii) poor adoption or late incorporation of genomic markers or improved (genomic) breeding technologies in breeding programs; (ix) and the scarcity of reliable markers that are diagnostic or closely linked to pre-harvest sprouting tolerance QTLs to expedite MAS.

Proposed solutions include the use of molecular markers and genomics in identifying pre-harvest sprouting tolerance traits. These techniques facilitate selection and are less sensitive to environmental conditions. Also, the adoption of improved genotypic breeding tools, such as speed-breeding technology, mutation breeding, genomic prediction, transgenic, and cisgenic breeding, as well as gene-editing breeding (CRISPR-Cas9), is important. The benefits of these technologies/tools include accelerated breeding cycles, increased genetic diversity, improved precision, and increased genetic gains. Genotypic breeding tools and technologies are effective independently, but their efficacy is enhanced when integrated with conventional breeding.

Breeding wheat for pre-harvest sprouting tolerance is ongoing in the South African wheat germplasm, with promising leads in the University of Stellenbosch, with funding received from the Department of Science and Innovation, the Technology Innovation Agency, and Grain SA. Improvement of pre-harvest sprouting tolerance in wheat is crucial to meet the Sustainable Development Goal 2.0 of ending all forms of hunger and malnutrition sustainably worldwide and promoting sustainable agriculture. Future research is anticipated to focus on the development of climate-resilient wheat varieties that are tolerant to pre-harvest sprouting, with the assistance of technological advancements, as well as collaborations and policy support. This is expected to improve food security and enhance economic sustainability in South Africa.

## Figures and Tables

**Table 1:** Pre-harvest sprouting tolerance status of the South African commercial wheat cultivars. Data taken from [48,54].

Production Region	Pre-Harvest Sprouting Tolerance in the Early 1990s	Status “Then”	Pre-Harvest Sprouting Tolerance Now	Status “Now”
Summer Rainfall	23%	Poor	>60%	Good–excellent
Irrigation	42%	Moderate	62%	Good
Winter rainfall	-	-	70–80%	Excellent

**Table 2:** Classification of South African wheat cultivars for pre-harvest spouting tolerance based on a scale of 1–8 that is used at the ARC-Small Grain to assess variation in sprouting on intact heads [36,45,54,62] (Figure 3).

Group	Scoring System	Characteristic(s)
Tolerant	1–3	Excellent response. None-to-minimal signs of sprouting, such as the emergence of few roots.
Moderate	3.1–4.5	Moderately tolerant or moderately susceptible response. Visible roots and signs of shoots. The reaction of these cultivars/genotypes is strongly influenced by environmental conditions rather than the genetic makeup, which makes them unstable across environments and years [24].
Susceptible	>4.5	Poor tolerance. Swollen, discolored, and heavily germinated kernels, fully grown roots and shoots (see Figure 1).

**Table 3:** Summary of isolated genes and their favorable alleles for improving pre-harvest sprouting resistance in wheat. The table is adopted from [112].

Genes	Chromosomes	Markers	Physical Site/Mb	Favorable Alleles	References
*TaVp-1*	3AL	Vp-1A	659.6	*TaVp-1Ab*, *TaVp-1Ad*	Chang et al., 2011 [115]
		Vp1A3		*TaVp-1Agm*	Yang et al., 2013 [119]
	3BL	Vp1-b2	693.3	*TaVp-1Bb*, *TaVp-1Bc*, *TaVp-1Bd*, *TaVp-1Be and TaVp-1Bf*	Chang et al., 2009a; Chang et al., 2009b [116,117]
		Vp1B3		*TaVp-1Bb*, *TaVp-1Bc*	Yang et al., 2007 [118]
*TaDOG1L1*	3AS	DOG1L1	534.5	*-*	Ashikawa et al., 2010 [120]
*TaMFT/TaPHS1*		MFT-A1		*TaMFT-A1b*	King & Richards, 1984 [67]
		MFT-A2		*TaMFT-3Aa*	Jiang et al., 2018 [124]
		MFT-3A	7.3	*SNP-222(C)*	Nakamura et al., 2011 [27]
		TaPHS1-SNP1/TaPHS1-SNP1		*SNP646/666 (G/A)*	Liu et al., 2013 [70]
*TaSdr*	2A	Sdr2A	158.5	*TaSdr-A1a*	Zhang et al., 2017 [125]

**Table 4:** Wheat wild relatives, landraces, and lines identified to have pre-harvest sprouting tolerance [66].

Wild Relatives/Landraces/Lines Identified with Pre-Harvest Sprouting Tolerance	Country	References
*Aegilops tauschii*	China	Deng-Cai et al., 1998 [158]
*Triticum Turgidum* and *Aegilops tauschii* (DD)	China	Deng-Cai et al., 1998; Yu et al., [158,159]
Synthetic hexaploid wheat genotypes (SHW-L1)	China	Yang et al., 2014 [119]
Chinese Landraces viz., Xiaoyuhua, Yongchuanbaike, and Baiyuhua	China	Xiao et al., 2002 [160]
Hongheshangtou and Wanxianbaimaizi	China	Yang et al., 2009; Chang et al., 2010 [116,161]
RL4137	Canada	Bassoi & Flintham 2005 [162]
BRS177	Brazil	Flintham 1993 [163]
Frontana	Brazil	Santos et al., 2010; Nornberg et al., 2015 [164,165]
RL4137	Canada	DePauw et al., 2009; DePauw et al., 2012 [139,140]
Konde, Kumpa, and Swindy cultivars	Australia	Jimenez et al., 2017 [166]

## Data Availability

The original contributions presented in this study are included in the article. Further inquiries can be directed toward the corresponding author.

## Supplementary Materials

The following supporting information can be downloaded at https://www.mdpi.com/article/10.3390/plants14142134/s1.

## Abbreviations

The following abbreviations are used in this manuscript:

ABA	abscisic acid
ARC-Small Grain	Agricultural Research Council—Small Grain Institute
COW	Class Other Wheat
CRISPR-Cas9	clustered regularly interspaced palindromic repeats-associated Cas-9 protein
CIMMYT	International Maize and Wheat Improvement Centre
DH	doubled-haploid
DNA	Deoxyribonucleic acid
DSI	Department of Science and Innovation
GA	gibberellic acid
GEBVs	genomic estimated breeding values
GP	genomic prediction
GWASs	genome-wide association studies
ICARDA	International Centre for Agricultural Research in the Dry Areas
ISPHSC	International Symposium on Pre-Harvest Sprouting in Cereals
KASP	Kompetitive allele-specific PCR
MAS	marker-assisted selection
MTA	marker–trait association
ND	no data available
PHS	pre-harvest sprouting
QTL(s)	quantitative trait locus/loci
SDG	Sustainable Development Goal
SNP	single-nucleotide polymorphism
SSR	simple sequence repeat
SU	Stellenbosch University
TIA	Technology Innovation Agency
USDA	United States Department of Agriculture
