# Supplementary material for: Breeding Wheat (Triticum aestivum L.) for Pre-Harvest Sprouting Tolerance in South Africa: Current Status and Future Prospects
Source: Plants (Basel). 2025 Jul 10;14(14):2134. doi: 10.3390/plants14142134 (PMC12299735; doi:10.3390/plants14142134)
Supplement: Supplementary file 1 [file plants-14-02134-s001.zip › plants-3711744-supplementary.pdf]

plants

MDPI

Table S1. Bread Wheat Grading Table [56].

Grade	Minimum			Maximum percentage permissible deviation (m/m)									
				A	B	C	D	E	F	G	H	I	J
	Hectolitre mass, kg/hl	Falling number, seconds	Protein content, %	Heavily frost damaged kernels	Field fungi	Storage fungi	Screenings	Other grain and unthreshed ears	Gravel, stones and turf	Foreign matter plus F	Heat damaged kernels	Damaged kernels plus H	Deviations (D + E + G + I) collectively
**Super Grade**	76	220	12.5	5	2	0.5	3	1	0.5	1	0.5	2	5
**Grade 1**	76	220	11.5	5	2	0.5	3	1	0.5	1	0.5	2	5
**Grade 2**	76	220	10.5	5	2	0.5	3	1	0.5	1	0.5	2	5
**Grade 3**	74	220	9.5	5	2	0.5	3	1	0.5	1	0.5	2	5
**COWǂ**	<74	<220	<9.5	>5	>2	>0.5	>3	>1	>0.5	>1	>0.5	>2	>5
**Minimum size of working samples**	1.5 kg unsifted	300 g cleaned	Apparatus instructions cleaned	25 g sifted	25 g sifted	100 g sifted	500 g unsifted	50 g sifted	100 g sifted	100 g sifted	100 g sifted	25 g sifted	-

ǂCOW, Class Other Wheat. This class does not meet standards for bread wheat and no grades are determined for COW.

Table S2. A summary of the major and stable QTL for pre-harvest sprouting/dormancy-related traits in wheat as adapted from 7D [18].

	Trait/QTL (PVE%)[a]	Linked marker	Physical position (Mbp)[b]	Environment	[c]References
1	FN/5A(26.4)	Xpsr1194–Xpsr918b	ND	2, Mean/4	Zanetti et al., 2000 [97]
2	α-AA/5A(30.0)	Xpsr1194–Xpsr918b	ND	3, Mean/4	Zanetti et al., 2000 [97]
3	SD/4AL(33–77.2)	Xcdo795/Xpsr115	ND	3/3	Kato et al., 2001 [98]
4	PHS/QPhs.ccsu-3A.1(78.3)	Xwmc153–Xgwm155	701.7–702.9	6, Mean/6	Kulwal et al., 2005 [99]
5	SD/QPhs.ocs-3A.1(23.0–44.8)	Xbarc310/Xbcd907	7.1	2/4	Mori et al., 2005 [100]
6	GI/QGi.crc-3B(27.0)	Xbarc77–Xwmc307	430.1–783.5	3, Mean/3	Fofana et al., 2009 [101]
7	SI/QSi.crc-3B(24.0)	Xbarc77–Xwmc307	430.1–783.5	3, Mean/3	Fofana et al., 2009 [101]
8	FN/QFn.crc-3B(33.0)	Xbarc77–Xwmc307	430.1–783.5	3, Mean/3	Fofana et al., 2009 [101]
9	GI-14/QPhs.dpivic-3D.1(26.0–43.0)	Red Grain Color RGC-wms1200	ND	2/4	Imtiaz et al., 2008 [80]
10	VI/QPhs.dpivic-4A.1 (21.0)	Xbarc170–Xgwm269c	605.7–607.8	2/4	Imtiaz et al., 2008 [80]
11	PHS/QPhs.pseru-3AS(31.26–44.96)	Xbarc12–Xbarc321	11.7-15.4	2, Mean/3	Liu et al., 2008 [102]
12	QPhs.dpi.vic.4A.2(27.78–39.84)	Xgwm637–Xgwm937	617.4	2, Mean/3	Ogbonnaya et al., 2008 [103]
13	PHS/2DS(25.73-–27.50)	Xgwm261–Xgwm484	19.6–48.1	2/2	Xiao-bo et al., 2008 [104]
14	GI/QGI.crc-4B(28.2–66.6)	Xwmc349	640.9	3, Mean/4	Rasul et al., 2009 [105]
15	PHS/QSI.crc-4B(6.2–26.9)	Xwmc349	640.9	2, Mean/5	Rasul et al., 2009 [105]
16	PHS/QPhs.cnl-2B.1(24.0)	Xbarc55–Xwmc474	133.5–172.6	16, Mean/16	Munkvold et al., 2009 [72]
17	GC/QGc.ccsu-3B.1(15.28–40.42)	Xgwm938–Xgwm980	ND	4, Mean/4	Kumar et al., 2009 [106]
18	PHS/QPhs.ccsu-6A.1 (12.01–29.47)	Xgwm1296–Xgwm1150	ND	3, Mean/4	Kumar et al., 2009 [106]
19	PHS/QPhs.caas-3AS.1 (11.8–27.7)	Xbarc294–Xbarc57	7.9-10.3	2, Mean/3	Miao et al., 2013 [107]
20	GI/QGi.crc-4A (27.6–58.1)	–	ND	3/3	Cabral et al., 2014 [34]
21	PHS(SI)/QSi.crc-4A (10.5–32.1)	–	ND	3/4	Cabral et al., 2014 [34]
22	PHS(SI)/QSi.crc-7B (11.8–20.5)	–	ND	1/2	Cabral et al., 2014 [34]
23	FN/QFn.crc-7D (13.2–20.6)	–	ND	1/2	Cabral et al., 2014 [34]
24	PHS, SD/Qphs.pseru-4A (17.2–26.5)	GBS_212432–GBS_109947	ND	2, Mean/5	Lin et al., 2015 [108]
25	QPhs.spa-4B (35.0–60.0)	Xwmc617b–Xwmc48a	15.7–98.7	7/7	Kumar et al., 2015 [109]
26	QPhs.spa-7D2 (14.0–47.0)	Xbarc76–Xcfa2257a	634.0	7/7	Kumar et al., 2015 [109]

27 GI/3AS (21.6–41.0)	KASP-222	7.2	3/3	Shao et al., 2018 [110]
28 qPHS.sicau-3D (8.65–42.47)	AX-94415259	562.5–5	7/9	Yang et al., 2019 [111]

index; GI, germination index; –, marker information not available; ND, physical position of QTL could not be determined due to lack of marker sequence information.

[a]Phenotypic variation explained, [b]Physical position of one flanking marker was given (instead of interval), if the second marker or its sequence was not available, [c]Environment = number of environments in which QTL was detected/number of total environments.

Table S3. Pre-harvest sprouting tolerance of South African wheat cultivars released between 1965 and 2014 by ARC-Small Grain, Syngenta Seed (formerly Sensako) and Corteva (formely Pannar Seed). The phenotypic pre-harvest sprouting tolerance class was confirmed using the SNP marker analysis (KASP markers TaPHS1-646 and TaPHS1-666) [36].

Dryland cultivars						
**Cultivar**	**Origin**	**Year released**	**# years evaluated**	**ǂMean PHS score ± SD**	**PHS class**	**§Predicted PHS class**
**Betta**	ARC-Small Grain	1969	4	1.5 ± 0.33	Tolerant	Tolerant
**Betta-DN**	ARC-Small Grain	1993	13	2.1 ± 0.92	Tolerant	Tolerant
**Caledon**	ARC-Small Grain	1996	15	2.7 ± 0.69	Tolerant	ND
**Elands**	ARC-Small Grain	1998	17	2.0 ± 0.71	Tolerant	Tolerant
**Gariep**	ARC-Small Grain	1994	18	3.5 ± 0.48	Tolerant	ND
**Karee**	ARC-Small Grain	1982	8	2.1 ± 0.69	Tolerant	Tolerant
**Komati**	ARC-Small Grain	2002	6	2.0 ± 0.41	Tolerant	Tolerant
**Letaba**	ARC-Small Grain	1987	3	3.2 ± 1.06	Tolerant	ND
**Limpopo**	ARC-Small Grain	1994	11	3.1 ± 0.94	Tolerant	Tolerant
**Matlabas**	ARC-Small Grain	2004	11	2.7 ± 0.56	Tolerant	Tolerant
**Molopo**	ARC-Small Grain	1988	3	3.2 ± 1.94	Tolerant	Tolerant
**PAN 3120**	Corteva	2002	11	2.6 ± 0.56	Tolerant	ND
**PAN 3144**	Corteva	2005	6	2.7 ± 0.48	Tolerant	Susceptible
**PAN 3355**	Corteva	2006	6	3.0 ± 0.49	Tolerant	Tolerant
**PAN 3364**	Corteva	1996	7	2.3 ± 0.82	Tolerant	ND

**PAN 3368**	Corteva	2007	7	2.4 ± 0.54	Tolerant	ND
**PAN 3377**	Corteva	1997	9	3.3 ± 1.03	Tolerant	ND
**Scheepers 69**	ARC-Small Grain	1969	2	2.0 ± 0.28	Tolerant	ND
**Senqu**	ARC-Small Grain	2010	4	2.7 ± 0.15	Tolerant	Tolerant
**SST 317**	Syngenta Seeds	2013	3	2.8 ± 0.06	Tolerant	ND
**SST 322**	Syngenta Seeds	2002	4	2.4 ± 0.54	Tolerant	ND
**SST 347**	Syngenta Seeds	2004	7	2.7 ± 0.6	Tolerant	ND
**SST 356**	Syngenta Seeds	2005	8	3.5 ± 0.36	Tolerant	ND
**SST 374**	Syngenta Seeds	2011	2	3.0 ± 0.85	Tolerant	ND
**SST 398**	Syngenta Seeds	2010	4	2.7 ± 1.04	Tolerant	Tolerant
**SST 399**	Syngenta Seeds	1999	7	2.8 ± 0.44	Tolerant	Tolerant
**SST 936**	Syngenta Seeds	1994	4	3.5 ± 0.39	Tolerant	ND
			**Irrigation cultivars**			
**Cultivar**	**Origin**	**Year released**	**# years evaluated**	**ǂMean PHS score ± SD**	**PHS class**	**§Predicted PHS class**
**Baviaans**	ARC-Small Grain	2000	11	2.9 ± 0.43	Tolerant	ND
**Biedou**	ARC-Small Grain	2001	1	2.9	Tolerant	Tolerant
**Buffels**	ARC-Small Grain	2007	6	2.5 ± 0.26	Tolerant	ND
**CRN 826**	ARC-Small Grain	2002	10	4.4 ± 0.59	Moderate	Susceptible
**Duzi**	ARC-Small Grain	2004	11	3.7 ± 0.38	Moderate	Tolerant

**Gamtoos**	ARC-Small Grain	1985	4	3.9 ± 0.99	Moderate	Susceptible
**Inia**	ARC-Small Grain	1970	9	4.1 ± 0.55	Moderate	Susceptible
**Kariega**	ARC-Small Grain	1993	17	2.5 ± 0.7	Tolerant	Tolerant
**Krokodil**	ARC-Small Grain	2004	11	4.1 ± 0.55	Moderate	ND
**Marico**	ARC-Small Grain	1993	12	3.1 ± 1.14	Tolerant	Susceptible
**Nantes**	ARC-Small Grain	1990	3	3.9 ± 0.89	Moderate	Susceptible
**Palmiet**	ARC-Small Grain	1985	6	4.4 ± 1.12	Moderate	Susceptible
**PAN 3400**	Corteva	2011	3	4.2 ± 1.05	Moderate	ND
**PAN 3434**	Corteva	2004	7	3.5 ± 0.55	Tolerant	Susceptible
**PAN 3478**	Corteva	2008	6	3.3 ± 0.3	Tolerant	Susceptible
**PAN 3497**	Corteva	2011	3	3.4 ± 0.35	Tolerant	Susceptible
**PAN 3515**	Corteva	2013	1	3.2	Tolerant	Tolerant
**PAN 3623**	Corteva	2013	1	2.5	Tolerant	ND
**Sabie**	ARC-Small Grain	2010	6	2.8 ± 0.56	Tolerant	Tolerant
**SST 38**	Syngenta Seeds	1993	6	2.9 ± 0.61	Tolerant	Tolerant
**SST 822**	Syngenta Seeds	1992	18	3.8 ± 0.91	Moderate	Susceptible
**SST 843**	Syngenta Seeds	2008	7	4.5 ± 0.6	Moderate	ND
**SST 866**	Syngenta Seeds	2011	5	4.0 ± 0.58	Moderate	ND
**SST 867**	Syngenta Seeds	2009	5	2.5 ± 0.44	Tolerant	Tolerant

**SST 875**	Syngenta Seeds	2012	5	4.3 ± 0.72	Moderate	ND
**SST 877**	Syngenta Seeds	2010	5	2.3 ± 0.28	Tolerant	Susceptible
**SST 884**	Syngenta Seeds	2013	4	4.7 ± 0.91	Moderate	Susceptible
**SST 895**	Syngenta Seeds	2014	4	3.2 ± 0.71	Tolerant	ND
**SST 896**	Syngenta Seeds	2014	1	5	Moderate	ND
**SST 33**	Syngenta Seeds	1988	3	4.5 ± 1.54	Moderate	Susceptible
**SST 86**	Syngenta Seeds	1988	2	3.3 ± 0.25	Tolerant	Tolerant
**T4**	ARC-Small Grain	1965	6	2.3 ± 0.8	Tolerant	Tolerant
**Tamboti**	ARC-Small Grain	2011	3	3.4 ± 0.32	Tolerant	Susceptible
**Timbavati**	ARC-Small Grain	2011	3	3.3 ± 0.85	Tolerant	Tolerant
**Umlazi**	ARC-Small Grain	2010	3	3.3 ± 0.23	Tolerant	ND

ǂPre-harvest sprouting scores of 1 – 3.5 are tolerant and scores of 3.5 – 4.5 are moderate.

§Pre-harvest sprouting class prediction according to the SNP marker analysis was either tolerant or susceptible (no moderate class).

ND, no data available.

Table S4. A set of breeding lines (doubled haploids (DH)) with notable pre-harvest sprouting tolerance in combination with good thousand kernel weight as identified by [62] and [63].

Entry no.	DH line†	Average PHS	Average TKW
Best performing DH lines with PHS score mean ≤ 2.00 and TKW 41.6 g			
21	TE 21	1.94	42.30
122	TE 135	1.51	44.50
Genotypes with PHS score mean ≤ 2.99 and TKW 40.00 g			
4	TE 4	2.71	46.92
6	TE 6	2.92	45.34
7	TE 7	2.73	41.50
8	TE 8	2.65	44.45
19	TE 19	2.85	40.43
21	TE 21	1.94	42.30
23	TE 23	2.47	46.16
24	TE 24	2.61	40.08
29	TE 29	2.97	49.95
32	TE 32	2.26	42.60
36	TE 36	2.98	40.50
37	TE 37	2.04	40.36
44	TE 45	2.36	46.67
46	TE 47	2.56	44.23
52	TE 54	2.46	45.15
53	TE 55	2.48	45.67
57	TE59	2.53	41.43
60	TE 62	2.01	40.28

Entry no.	DH line†	Average PHS	Average TKW
Best performing DH lines with PHS score mean ≤ 2.00 and TKW 41.6 g			
146	EF 9	1.94	42.16
179	EF 44	1.44	51.60
Genotypes with PHS score mean ≤ 2.99 and TKW 40.00 g			
75	TE 79	2.12	41.90
84	TE 90	2.65	40.43
91	TE 97	2.40	47.50
94	TE 101	2.78	43.13
98	TE 105	2.51	45.46
103	TE 113	2.90	42.32
105	TE 115	2.60	41.78
107	TE 118	2.63	42.70
115	TE 127	2.30	42.48
116	TE 128	2.73	40.78
119	TE 131	2.97	43.00
120	TE 132	2.57	41.20
122	TE 135	1.51	44.50
123	TE 136	2.43	46.50
126	TE 141	2.94	43.67
127	TE 142	2.26	41.56
137	TE 153	2.94	49.87
140	EF 1	2.22	43.46

Entry no.	DH line†	Average PHS	Average TKW
Best performing DH lines with PHS score mean ≤ 2.00 and TKW 41.6 g			
180	EF 45	1.89	42.98
Genotypes with PHS score mean ≤ 2.99 and TKW 40.00 g			
168	EF 31	2.43	41.18
172	EF 37	2.83	44.18
175	EF 40	1.97	41.27
179	EF 44	1.44	51.60
180	EF 45	1.89	42.98
181	EF 46	2.23	40.94
183	EF 48	2.22	46.33
190	EF 56	2.75	40.40
192	EF 58	2.57	41.83
141	EF 3	2.72	40.13
144	EF 7	2.05	41.28
146	EF 9	1.94	42.16
153	EF 16	2.55	45.40
159	EF 22	2.06	40.05
168	EF 31	2.43	41.18
172	EF 37	2.83	44.18
175	EF 40	1.97	41.27
179	EF 44	1.44	51.60

61	TE 63	2.85	46.67	141	EF 3	2.72	40.13	180	EF 45	1.89	42.98
68	TE 71	2.49	45.50	144	EF 7	2.05	41.28	181	EF 46	2.23	40.94
70	TE 74	2.23	40.32	146	EF 9	1.94	42.16	183	EF 48	2.22	46.33
72	TE 76	2.18	44.43	153	EF 16	2.55	45.40	190	EF 56	2.75	40.40
74	TE 78	2.79	45.60	159	EF 22	2.06	40.05	192	EF 58	2.57	41.83
Parents (checks)				Parents (checks)				Parents (checks)			
	Tugela-Dn	5.00	44.10		**Elands**	**2.00**	**41.60**		Flamink	3.00	40.50

†DH line: TE denotes a Tugela-Dn/Elands line and EF denotes an Elands/Flamink line. The parent in bold, Elands, was used as a selection standard.
